# Supplementary figures and images for: Early visual signatures and benefits of intra-saccadic motion streaks
Source: PLoS Comput Biol. 2025 Sep 29;21(9):e1013544. doi: 10.1371/journal.pcbi.1013544 (PMC12507291; doi:10.1371/journal.pcbi.1013544)

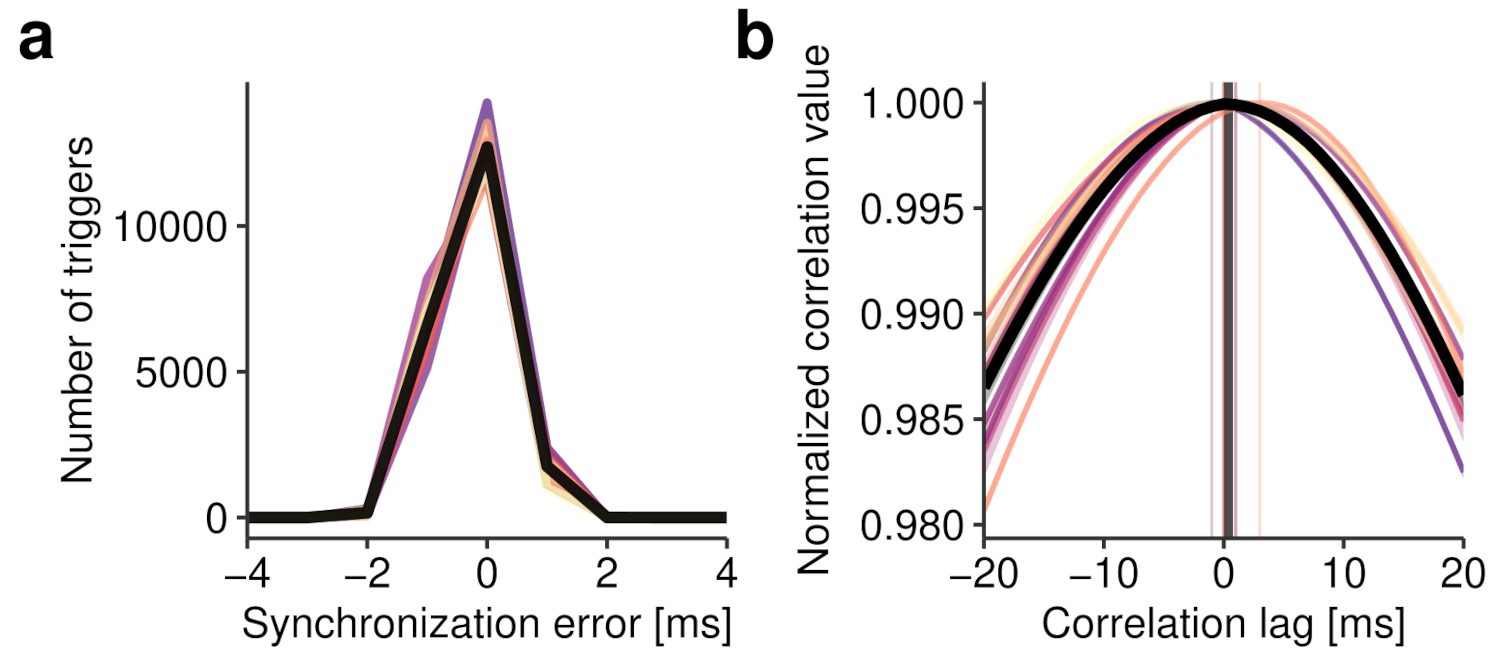

Supplement: S1 Fig — a Distribution of trigger sample lags when temporally aligning EEG and eye-tracking data after each signal was downsampled to 1000 Hz. b Results of cross-correlating EOG and eye-tracking eye position estimates (according to [20]) to check the alignment of both recordings. Positive lags would indicate that eye-movement events occur earlier in EOG than in eye-tracking data. In both panels, individual observers are shown as colored lines and solid black lines represent grand averages. (TIFF) [file pcbi.1013544.s001.tif]

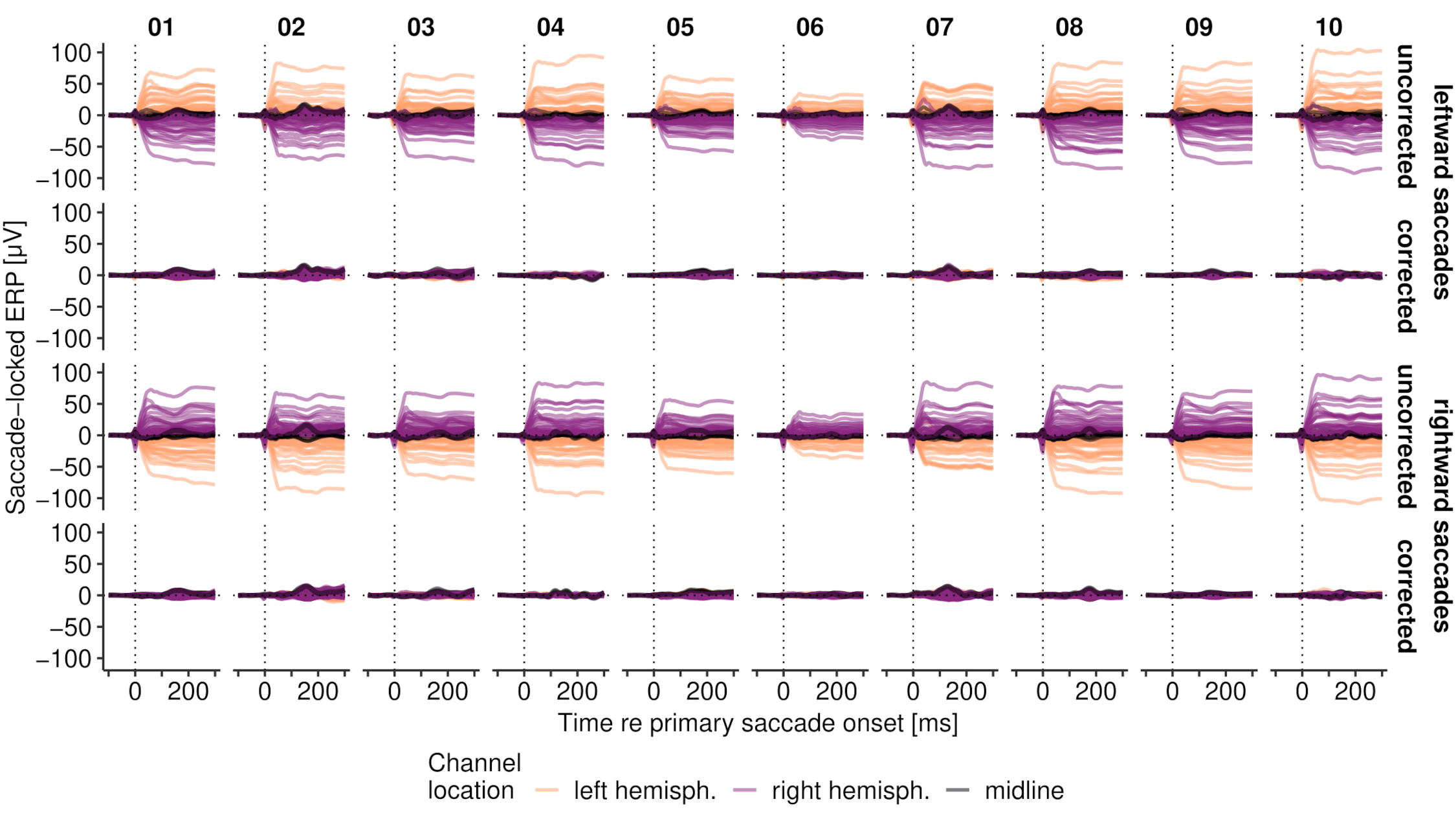

Supplement: S2 Fig — Panels show each observer’s data (all channels, except for EOG and mastoid electrodes) around the time of leftward (upper) and rightward (lower) primary saccades, before and after rejecting critical ICA components (see Sect 5.7.2). (TIFF) [file pcbi.1013544.s002.tif]

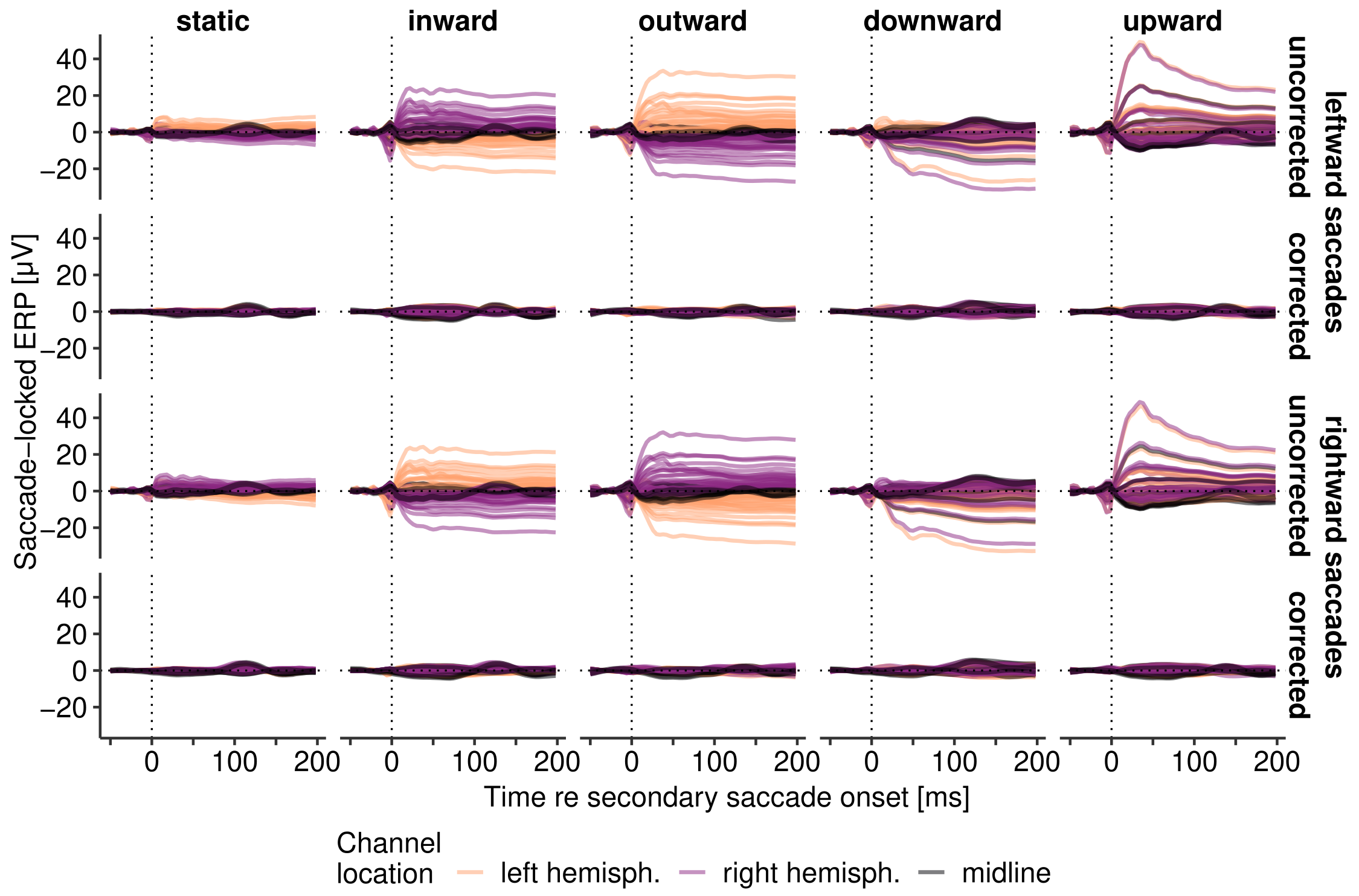

Supplement: S3 Fig — (TIFF) [file pcbi.1013544.s003.tif]

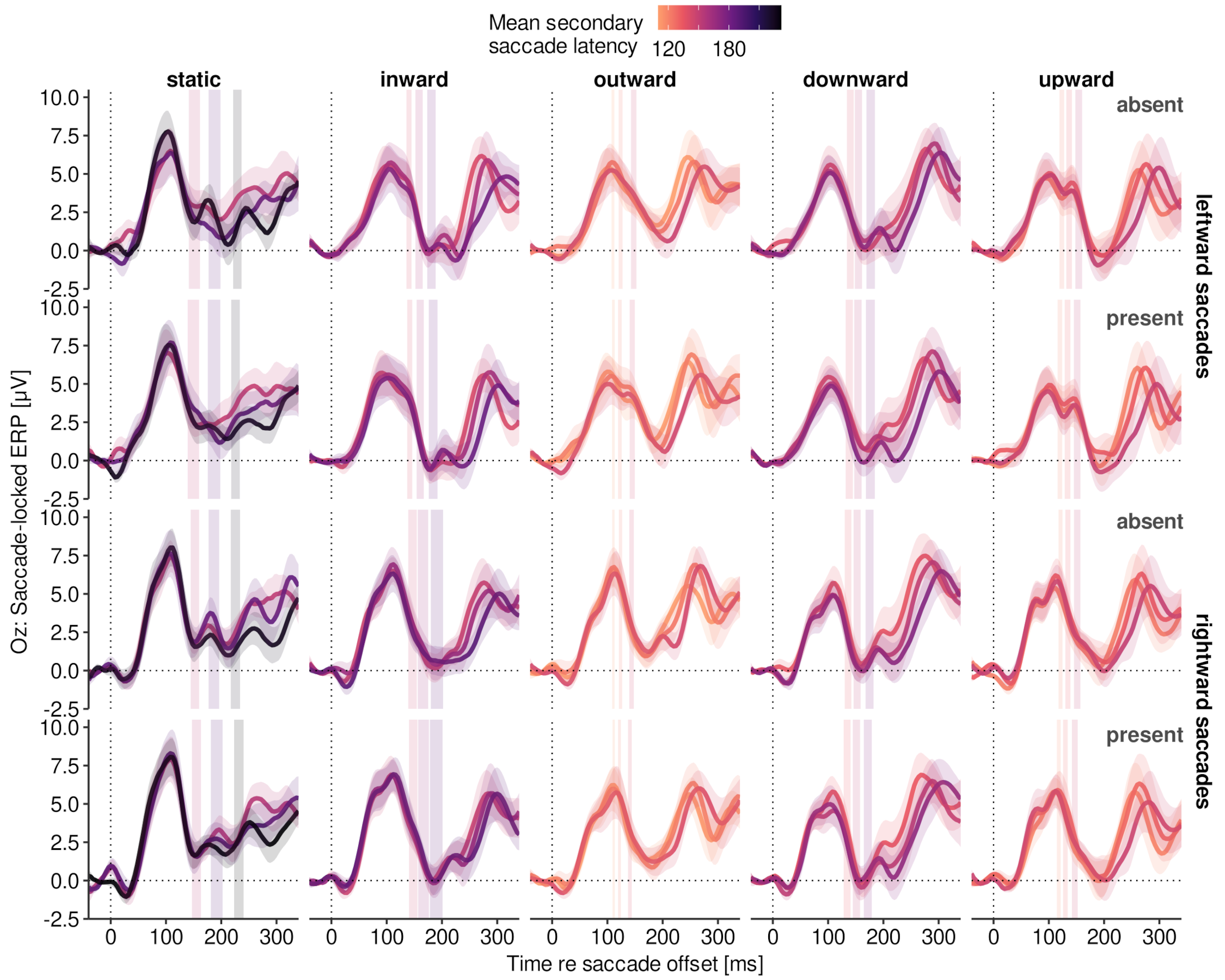

Supplement: S4 Fig — Average fixation-related potentials recorded from electrode Oz (±1 SEM), or λ waves, are shown for all experimental conditions (columns: target motion direction; rows: primary saccade direction × continuous target motion) and for three secondary saccade-latency bins, each containing the same number of trials in a given experimental cell. Vertical shaded areas indicate each condition’s and bin’s mean onset of the secondary saccade ±1 SEM. (TIFF) [file pcbi.1013544.s004.tif]

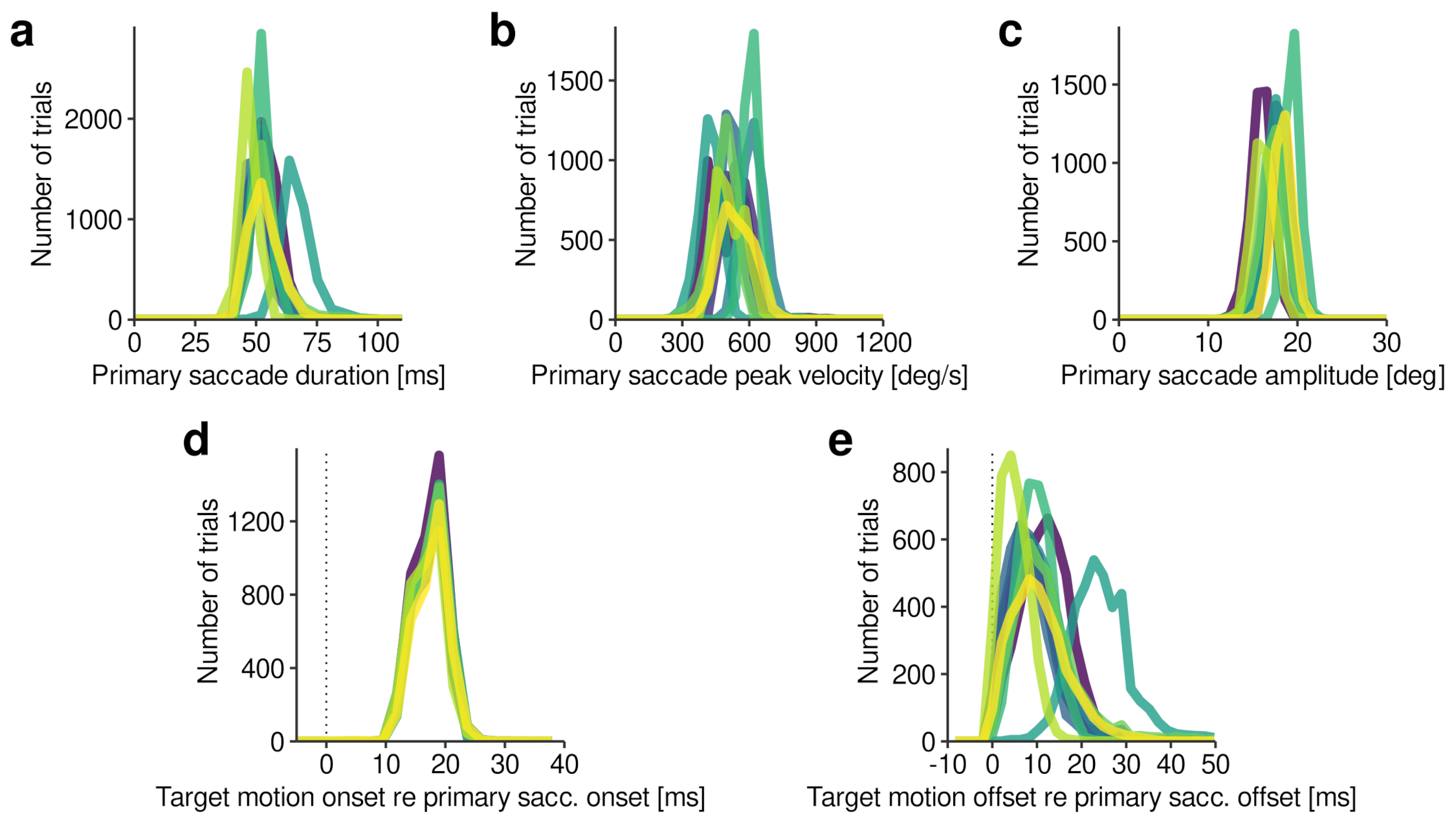

Supplement: S5 Fig — Individual observers are color-coded. (TIFF) [file pcbi.1013544.s005.tif]

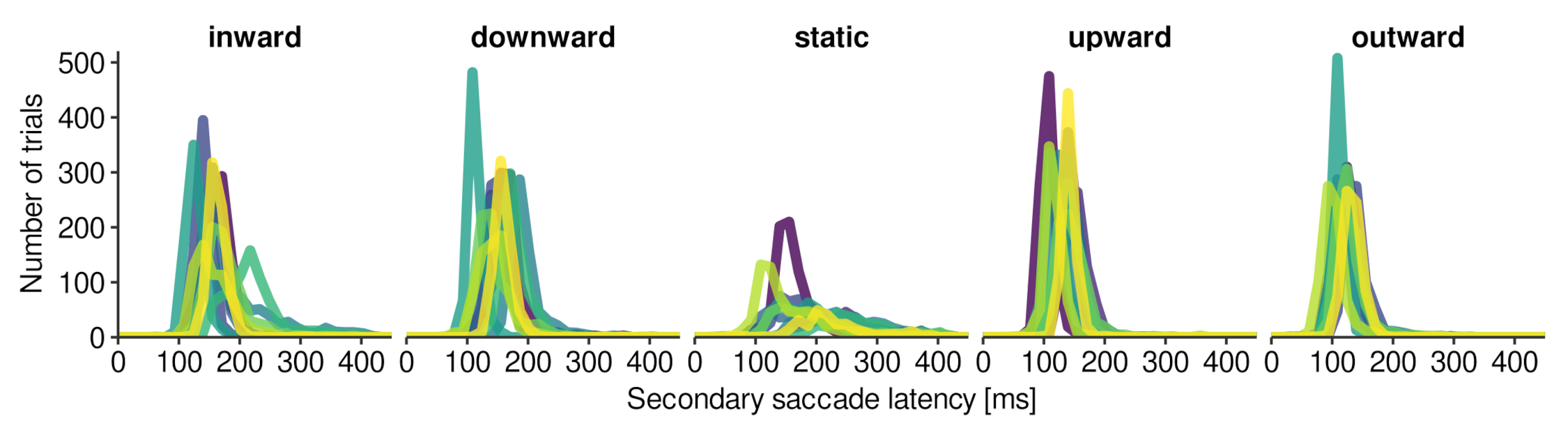

Supplement: S6 Fig — Individual observers are color-coded. (TIFF) [file pcbi.1013544.s006.tif]

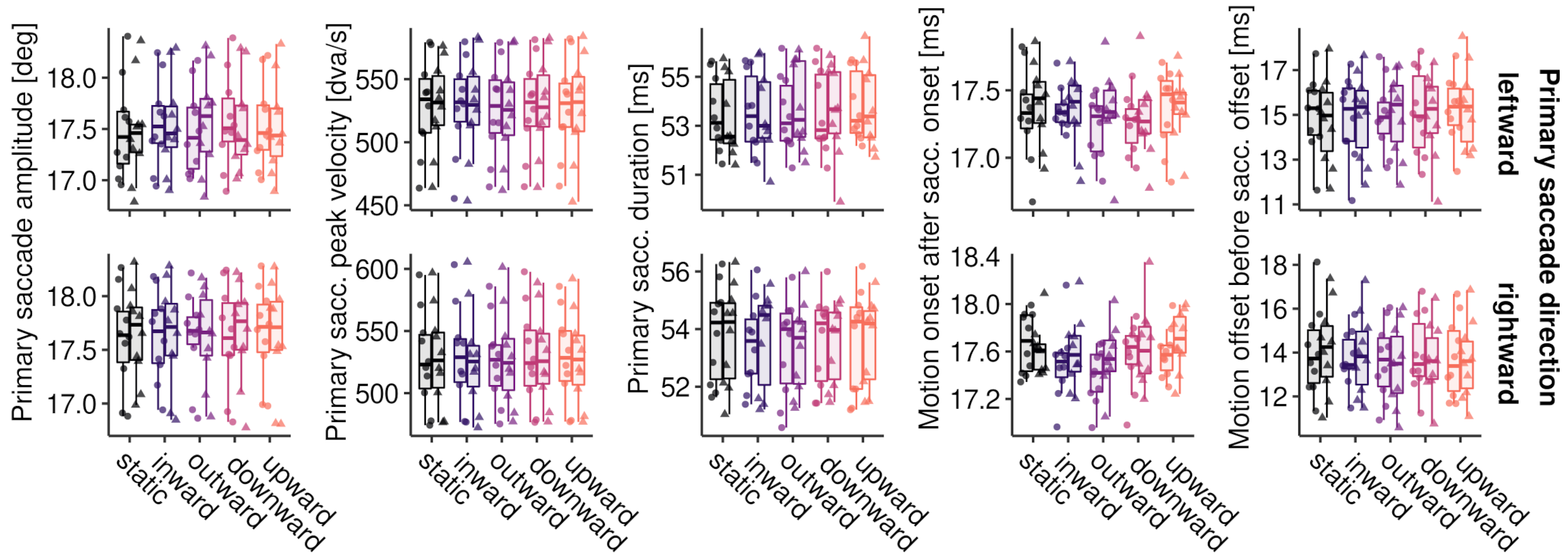

Supplement: S7 Fig — Left boxplots and circle dots indicate the motion-absent condition, whereas right boxplots and triangle dots indicate the motion-present condition. (TIFF) [file pcbi.1013544.s007.tif]

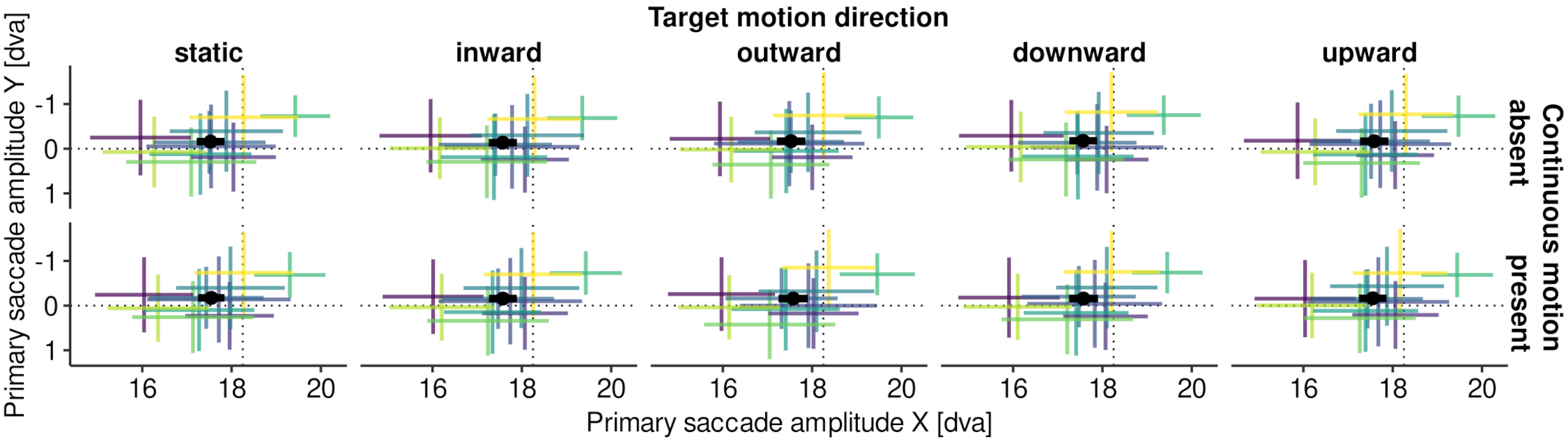

Supplement: S8 Fig — Dotted lines represent instructed saccade amplitude whereas semi-transparent colored crosses indicate the mean amplitude component on each dimension (±1 SD) for individual observers in the experimental conditions target motion direction (columns) and continuous motion (rows). Black crosses show their respective population mean ±1 SEM. (TIFF) [file pcbi.1013544.s008.tif]

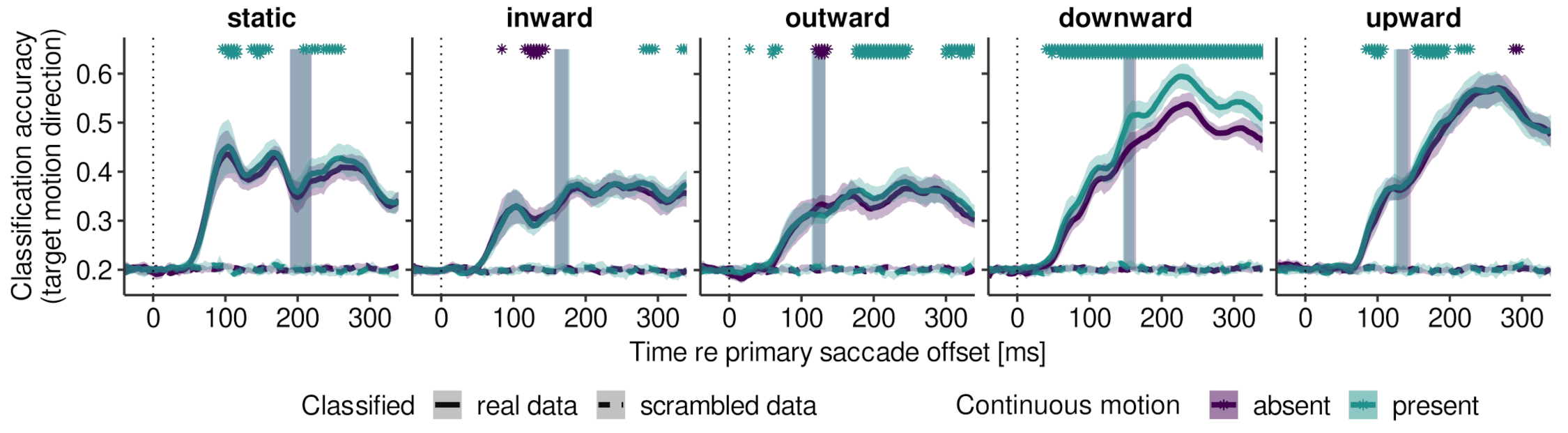

Supplement: S9 Fig — Classifiers were trained on data in absent conditions and then subsequently used to predict motion direction in absent and present conditions. See Fig 2 for figure conventions. (TIFF) [file pcbi.1013544.s009.tif]

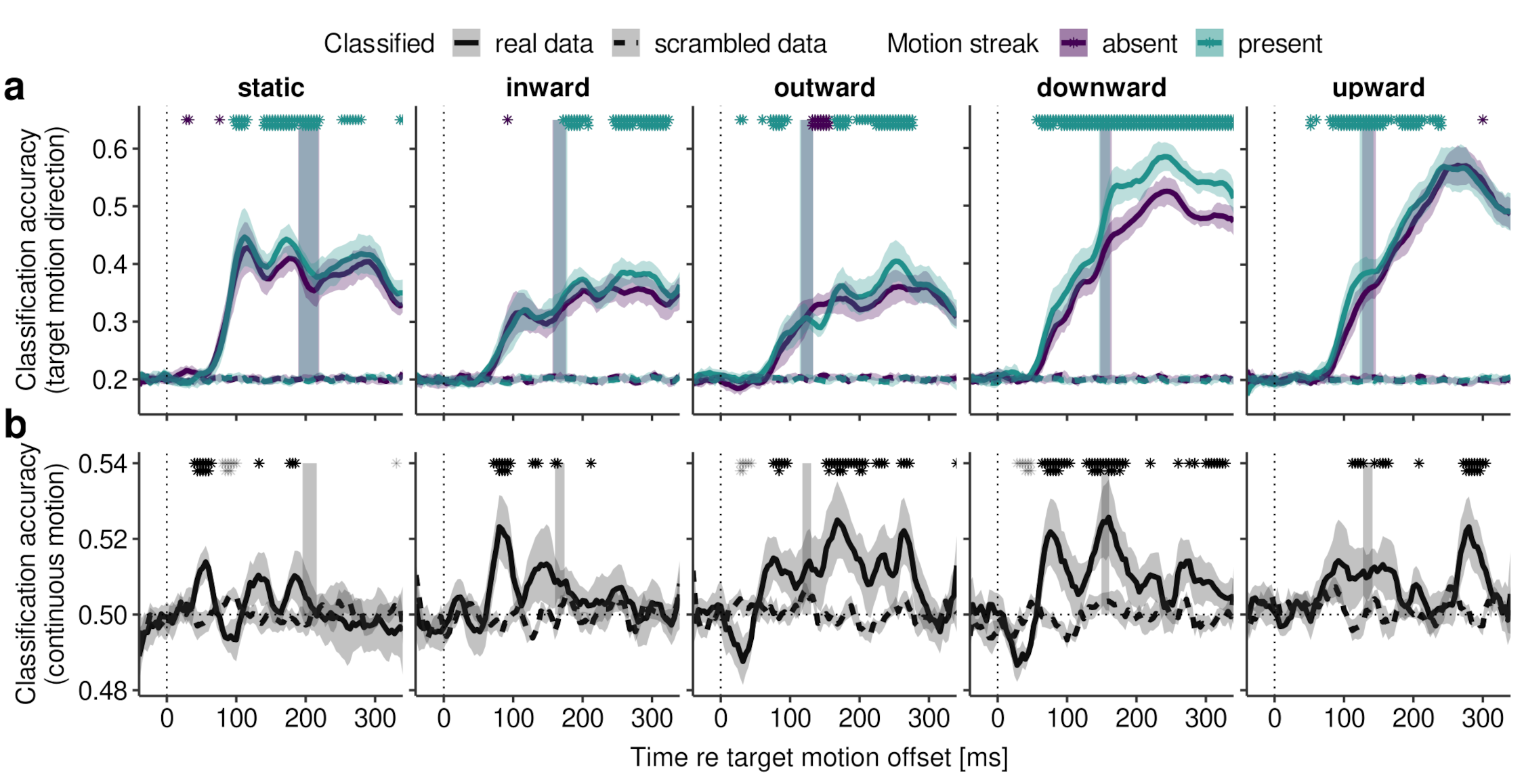

Supplement: S10 Fig — Same as Fig 2b and 2c, only that all analyses were performed relative target motion offset (vertical dotted line at 0 ms), instead of saccade offset. (TIFF) [file pcbi.1013544.s010.tif]

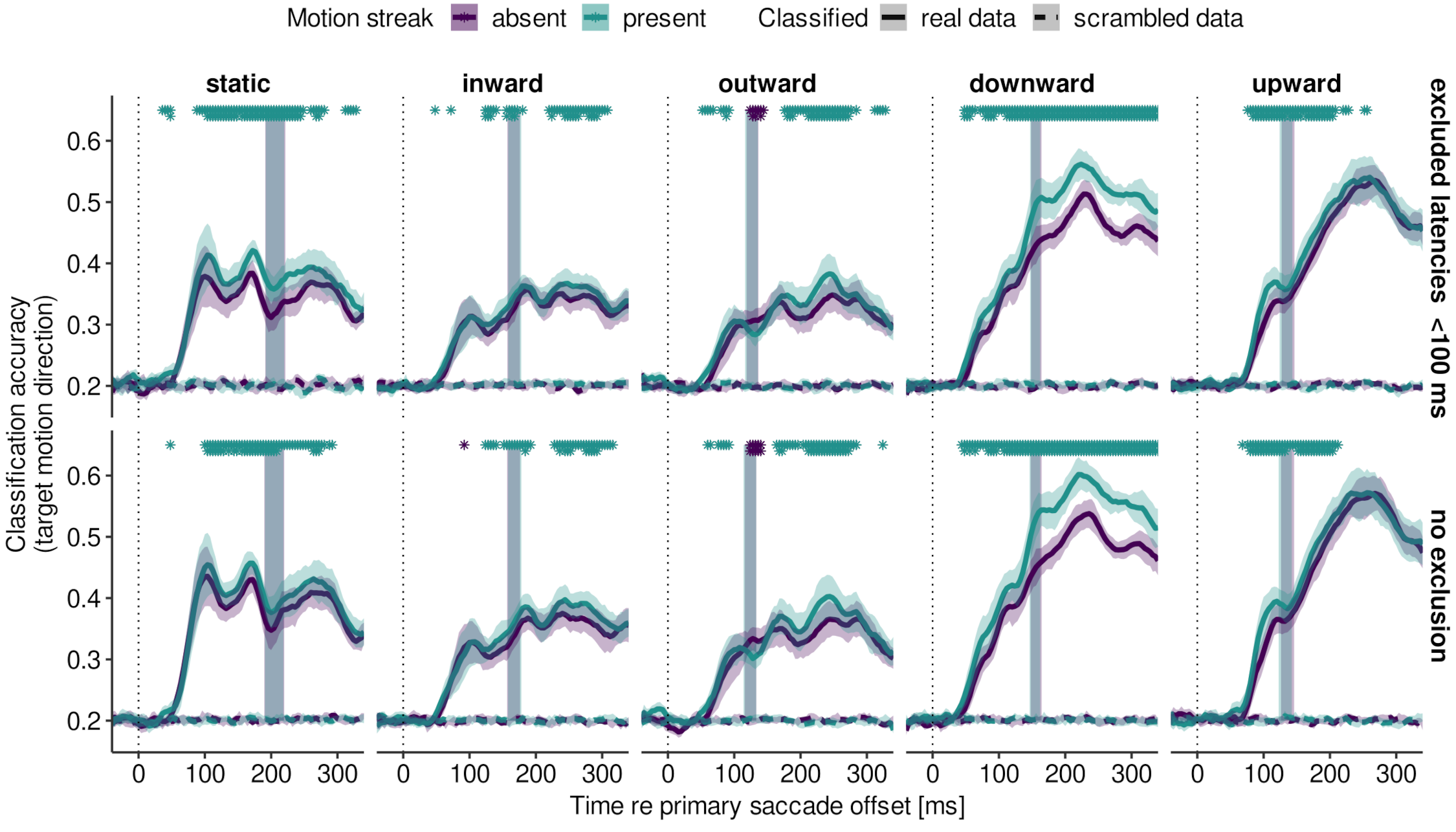

Supplement: S11 Fig — First-row asterisks denote significance levels of cluster-based permutation tests comparing present and absent conditions with <.05 (first row) and <.01 (second row). (TIFF) [file pcbi.1013544.s011.tif]

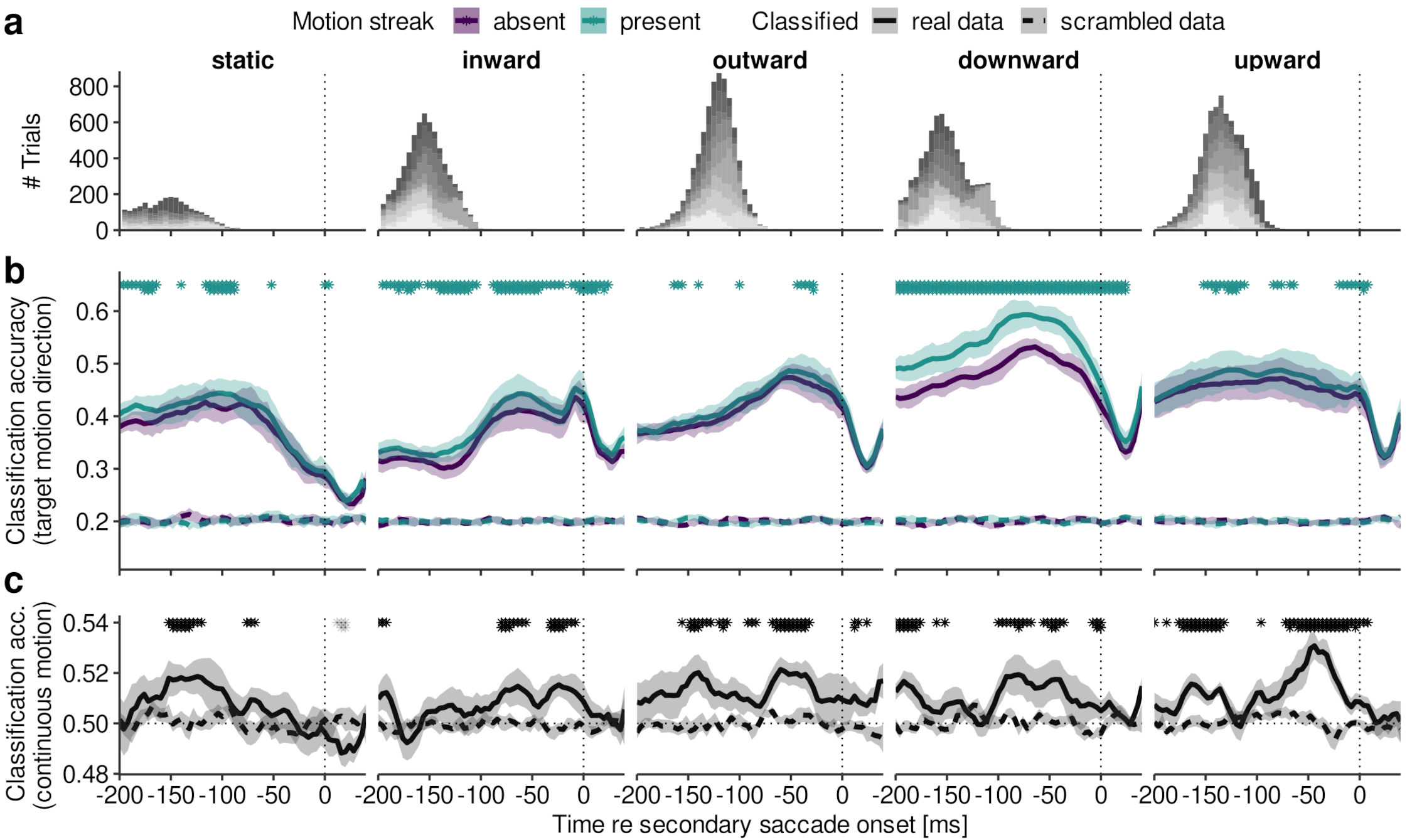

Supplement: S12 Fig — a Distribution of primary saccade offsets. Shades represent different observers. b Accuracy of classifying target motion direction, separately for present and absent conditions. Asterisks indicate results of cluster-based permutation tests comparing these two conditions. c Accuracy of classifying present vs absent motion conditions. Cluster-based permutation tests compared classification performance for real and scrambled class labels. All shaded error bars indicate ±1 SEM. First-row asterisks denote a significance level of <.05, whereas second-row denote <.01. Dashed lines show baseline classification accuracy, computed by performing the decoding procedure with scrambled class labels. (TIFF) [file pcbi.1013544.s012.tif]
