# Supplementary material for: Early visual signatures and benefits of intra-saccadic motion streaks
Source: PLoS Comput Biol. 2025 Sep 29;21(9):e1013544. doi: 10.1371/journal.pcbi.1013544 (PMC12507291; doi:10.1371/journal.pcbi.1013544)
Supplement: Attachment — Submitted filename: SchweitzerSeelRaischRolfs_2025_rev1_response.pdf [file pcbi.1013544.s013.pdf]

Reviewer 1

R1.General Thank you for the opportunity to provide a review of the manuscript entitled “Early visual signatures and benefits of intra-saccadic motion streaks” by Schweitzer and colleagues.
In the manuscript, the authors report an interesting combined EEG/eyetracking study in which a high-temporal resolution display was used to tightly control stimulus presentation during a saccade, whilst the eyes were in motion. By moving the saccade target either smoothly or abruptly (after a 25ms delay), the authors elicited secondary saccades, and compared the properties of those secondary saccades across smooth/jump conditions. In addition, they used EEG decoding to investigate how neural signatures of target position evolved during this period.
Overall, the manuscript is well-written, with a well-designed experiment and a strong theoretical underpinning. I am overall sympathetic to the conclusions drawn from the study, and the results neatly fit with previous work by the same authors and complement those earlier conclusions.

Response We are grateful for the reviewer’s overall positive evaluation of our work.

R1.P1 My main concern is a methodological issue related to the interpretation of one of the EEG analysis approaches. Specifically, the authors train classifiers to decode the position of the final target position (1 out of 5 possible positions) and, then test this on saccade trials, contrasting a smooth movement condition where the target shifts smoothly to the new location vs a “jump” condition where the target abruptly disappears and reappears in the new location.
This introduces a confound for the unfortunate reason that during the 25 ms gap, there is contrast energy on the screen in one condition that is not there in the other condition. Importantly, the location of that contrast energy (naturally) directly corresponds to the future location of the reappearing object (since it is moving on its way there). As such, it is unsurprising that it is systematically informative about the future position of the object – not necessarily because that information is used in a neural computation, but merely because those trials provide earlier visual input that “looks like” the to-be-decoded target location. The finding that decoding performance ramps up earlier in the smooth motion condition (e.g. Fig 7b) is therefore unsurprising – this is expected, simply because there is information on the screen earlier in that condition, which is visually (and therefore neurally) similar to what the classifier is trained to discriminate (position). In the jump condition, there is no visual information available until reappearance, so decoding performance naturally cannot rise. I therefore don’t think we can really draw an important conclusion from this result.
Ultimately I don’t think this is a huge problem, because the more interesting finding (to me) is that the orientation decoding works – even generalizing across motion directions (and therefore starting and ending positions). Although the same problem holds (there is something informative on the screen in one condition before anything becomes available in the other condition), the fact that it generalizes across direction is, to me, a stronger demonstration that the streak is indeed encoded as orientation, and that that information is available to be used to accelerate subsequent processing.

Response We thank the reviewer for bringing up this important point. First, it is correct that during the (target location-contingent) 25-ms period of target motion contrast energy is either present or absent. This choice was deliberate and necessary for investigating the question whether the target’s motion (or motion-streak) energy plays a role at all – that is, one could well assume that visual signals induced during saccades are proactively suppressed or removed from processing, e.g., as early as at the LGN, as previously suggested (Burr et al., 1994). In such a case, where the stimulus is not processed at the level of V1, one would not expect an earlier ramp-up of decoding accuracy with the topography that we find. In addition, previous psychophysical measurements also suggested that motion streaks could be perceptually discriminated from jump movements with hardly above-chance accuracy (Schweitzer and Rolfs, 2020). In this sense, we disagree with the reviewer’s view that one cannot ”draw an important conclusion from this result”: The finding of earlier decoding of streaky motion compared to step-like motion suggests than the contrast energy of the stimulus presented during that brief interval is indeed picked up and processed in early visual cortical areas.
Second, and more importantly, the reviewer is rightfully concerned whether this neural signature is actually ”systematically informative about the future position of the object”. We agree that, due to the fact that classifiers in the motion-present condition were able to incorporate streak-related signals whereas classifiers in the motion-absent condition were not, any interpretation regarding post-saccadic target location could be compromised. To rule out this possibility, we had – already in the initial version of the manuscript – performed an additional decoding analysis (see Appendix C, Figure C1) in which

we had trained classifiers exclusively on motion-absent trials, so that they could not learn from ”visual input that ’looks like’ the to-be-decoded target location”. Results of this control analysis were largely similar to the results achieved with the original decoding procedure, suggesting that the earlier ramp-up of decoding accuracy in the motion-present condition could in fact not be attributed merely to the fact that classifiers were also trained on motion-present trials – motion-present trials still yielded a benefit when decoding post-saccadic target location, even though classifiers had never ’seen’ any continuous intra-saccadic target motion.
We hope that this analysis and argument is convincing and have accordingly extended the paragraph in the paper that discusses these findings:

> The early onset and improvement of decoding accuracy arising from the presentation of continuous (as opposed to step-like) target motion during saccades suggests that these signals were not preemptively removed from visual processing, for instance, as early as in the LGN (Burr et al., 1994), but that they elicited unique cortical activation patterns that classifiers were well able to extract. It is however less straightforward to answer whether these patterns were systematically informative about the post-saccadic location of the target. In fact, it could be argued that it would only be natural to find earlier decoding of the target’s location in the case of continuous motion: Since motion was always contingent upon the to-be-decoded post-saccadic target location, classifiers could have learned from the neural responses to the target motion presented during the 25-ms interval, whereas in the motion-absent condition they could not. To rule out the possibility that the systematic differences between motion-present and motion-absent conditions were merely an artifact of classifying post-saccadic target location separately in these conditions, we devised a generalization test in which we trained classifiers exclusively on motion-absent trials, so that they could not have picked up the target’s streaky motion specifically, and then let them predict post-saccadic target location in motion-present trials. As shown in Figure C1, classifiers trained exclusively on motion-absent trials exhibited improved accuracy when predicting motion-present trials (M = 0.530, SD = 0.070) compared to when predicting motion-absent trials (M = 0.514, SD = 0.068; $F(1, 9)$ = 66.81, η^2 = 0.009, $p < .001$), and again this improvement did not occur similarly across all target motion directions ($F(2, 18) = 4.12$, $\eta^2 = 0.004$, $p = .035$, $p_{GG} = .044$). Before secondary saccades were made, classification of target movement orthogonal to primary saccade direction was significantly improved (M = +1.9%, $t(9)$ = 3.00, p = .015), classification of static targets improved with only marginal significance (M = +2.1%, $t(9)$ = 2.22, p = .053), whereas parallel target movement clearly did not elicit any improvement ($M = -0.6\%$, $t(9) = -1.19$, $p = .261$). The generalization test thus confirmed results up to this point – despite the classifiers’ inability to learn from continuous target motion. This suggests that early-onset, improved classification accuracy was not an artifact of the stimulus or classification procedure used, but was most likely related to a strengthened direction signal: To some extent, the visual signal resulting from continuous motion must have given away the post-saccadic target location, allowing the classifier to extract this pattern earlier than it would have otherwise been able to.

R1.P2 Minor point: the paper is overall well-written, but I note that the paper seems to almost deliberately avoid the topic of saccadic remapping. I understand that remapping is distinct from intrasaccadic perception, but particularly given the methodological approach of using EEG decoding during this period, it seems relevant to discuss recent work using similar approaches, including Fabius et al in JNeuro (10.1523/JNEUROSCI.1169-20.2020) and Moran et al JNeuro (10.1523/JNEUROSCI.2134-23.2024; particularly relevant because position was decoded, like in the current manuscript).

Response Again, we thank the reviewer for pointing this out. We did not ”deliberately avoid the topic of saccadic remapping”, but found that our study had little relevance for understanding the mechanisms underlying remapping. However, we agree that the methodological similarity to these studies may help to put our experimental approach into context. We have thus added the following paragraph:

> Capitalizing on the high temporal resolution of EEG to investigate the temporal dynamics of cortical activity that arose from such streak patterns, our study critically depended on decoding analyses. MVPA has successfully been applied to the investigation of saccadic

remapping, that is, the process by which neurons predictively respond to stimuli that will be in their RF upon landing of the impending saccade (Duhamel et al., 1992). For example, classifiers were trained on neural responses to different stimulus categories (such as faces vs houses or gratings with CW vs CCW orientation) viewed during fixation and then tested at various time points around the saccade, revealing the time course of information transfer across saccades (Edwards et al., 2018; Fabius et al., 2020). Other studies tested the spatial specificity of remapping, training classifiers to detect the presence of a stimulus at different screen locations, and then determining when before a saccade remapped activity became available if the location corresponded to the stimulus' future (post-saccadic) retinotopic location (Moran et al., 2024). Due to the strictly intra-saccadic stimulus presentations we applied, our study cannot make a statement about remapping. Yet, our experimental approach pursued a similar goal, that is, to determine at what time and processing stage critical neural signatures – in our case, of motion streaks – first emerge. Importantly, even though motion streaks during saccades were task-irrelevant and perceptually hardly noticeable (Schweitzer and Rolfs, 2020), decoding of not only their presence, but also their orientation was possible within less than 100 ms after saccade offset in every motion-direction condition. Corroborated by their occipital topography, these results suggest that intra-saccadic visual signals are processed together with the visual image of the new fixation – and from there could facilitate the programming of visually guided secondary saccades.

Reviewer 2

R2.General This is an excellent study, using concurrent EEG and eye tracking to examine the perception of continuous motion during saccadic eye movements. Although previous behavioral findings from this group are already quite compelling, the present study adds to these findings by suggesting that the source of the effect lies in the visual cortex and offers more detailed temporal information on its dynamics. Then, they develop a computational model that sheds new light on the physiological correlates of this perceptual phenomenon. The model supports the hypothesis that the target's motion trajectory is coded by orientation-selective circuits.
The study is well designed and includes thorough, state-of-the-art analyses that are, without doubt, well thought out. The findings are interesting and robust. I support the publication of this paper, but I also have a few concerns that should be addressed before it is published.

Response We thank the reviewer for their positive and constructive evaluation of our manuscript.

R2.P1 First, a general comment on the writing of the paper. The manuscript is highly impressive in terms of the analyses, the computational model, and the depth of thought evident in every detail. However, the writing could be clearer and more communicative. I found it very difficult to navigate the paper and locate answers to some of my questions. Even though the study overlaps significantly with my own field of expertise, I found it difficult to read and understand. I'm concerned that, for the broader readership of PLOS Computational Biology, this would be an almost impossible task.

Response We thank the reviewer for this feedback. As the reviewer did not make specific suggestions how to make the paper "clearer and more communicative", we made a series of edits and additions to the main text that should improve its perspicuity and readability. As these changes are too many to be included in this point-to-point reply and may appear out of context, they are best tracked in the revised manuscript. The following changes were made to the manuscript:

- Extension of the Introduction to more clearly formulate the research question and the goal of the study
- Strengthening of the narrative arc by outlining the three sequential levels of analysis (behavioral, physiological, computational) at the end of the Introduction
- Enhancement of the consistency in terminology across the entire manuscript
- Improved structuring of the section 'Decoding of the EEG signal' by outlining the three major decoding analyses performed

- Revision of paragraph structure to improve segmentation of individual trains of thought, as well as coherence across the entire manuscript
- Addition of introductory sentences to Results paragraphs to establish context to previous findings and improve readability

R2.P2 My second concern is methodological. The authors argue that they find a significant difference in classification performance between the two conditions (absent and continuous motion) during the short period between the offset of the first saccade and the onset of the second. The offset of the first saccade is well defined per trial (as the classification is segmented relative to this offset), but the timing of the second saccade is not well considered. The authors claim that the effect exists prior to the onset of the second saccade, but in fact, they did not check the second saccade onset in individual trials and (as far as I understand) not even in individual participants. Instead, they rely on the average time of the second saccade across participants. Since saccade onsets are highly variable and follow an ex-Gaussian distribution, it is likely that the earliest secondary saccades (which fall at the beginning of the distribution's rising slope) occur much earlier than the average.
This is especially problematic because the oculomotor and visual activity that accompanies saccades in the EEG is highly correlated with the direction of the saccade. Classification is highly sensitive to informative data and can therefore be significantly affected by even a small number of saccades. In other words, even a few secondary saccades that occur earlier than the rest may aid classification and lead to misinterpretation. Moreover, the findings show a behavioral difference in saccade onsets between the conditions, with real motion occurring earlier than apparent motion. This again raises the question of whether the EEG effects stem from differences in independent visual activity or are the direct result of eye movements.
A possible and (relatively) easy solution could be to exclude all trials with saccades in the early time range (e.g., until 100 ms), and then focus the analysis only on that window.

Response We thank the reviewer for pointing out this important potential confound and share their view that secondary saccades should have been better controlled for. Note that we had already confirmed our results using "only time points prior to each observer's mean secondary saccade onset in a given condition" (l. 88 in the initial manuscript). But we agree that this control may not have been rigorous enough. To remedy this shortcoming, we performed two additional analyses.
First, as suggested by the reviewer, we re-ran decoding analyses without trials with secondary sacccade latencies shorter than 100 ms, added these results to the Appendix (Figure C3) of the manuscript, and discuss them in the main text as follows:

> In order to make sure that low-latency secondary saccades – which may well have occurred earlier than each observer's estimated mean secondary saccade latency – were not the primary cause of this improvement, we excluded all trials with secondary saccade latencies below 100 ms, that is, on average 3.8% of trials (*SD* = 4.7), and reran the decoding of post-saccadic target location. Results of this control analysis were virtually identical to those reported above (for a comparison, see Figure C3), confirming that the earliest improvements in decoding accuracy were not merely the result of early eye movements.

Second, we performed decoding analyses on a time window prior to each trial's onset of the secondary saccade. The idea behind this analysis is that successful decoding considerably before secondary saccades are initiated is unlikely caused by oculomotor activity. This analysis was not included in the first version of the manuscript because the broad distribution of secondary saccade latencies led to a large amount of temporal smearing across the interval of interest. Yet, it should provide a definitive test that indeed "the effect exists prior to the onset of the second saccade". We have added this figure to the Appendix (Figure C4), too, and added the following to the main text:

> As, arguably, an occipital topography cannot rule out the involvement of oculomotor activity in driving decoding accuracy, especially given the close temporal proximity between post-saccadic visual responses and the initiation of secondary saccades, we classified post-saccadic target locations and the presence of continuous intra-saccadic motion in a time window that strictly preceded the onset of each trial's secondary saccade. Despite temporal smearing introduced by the variance in secondary-saccade latencies, this additional

> control analysis revealed that both variables could be reliably decoded (even long) before secondary saccades were initiated (Figure C4), further corroborating the notion that differences in visual activity, not in eye movements, allowed classifiers to differentiate between experimental conditions.

R2.P3 I also have a few minor comments:
1. I think it is important to show not only the decoding outcome but also the ERP (the lambda wave).

Response We agree with the reviewer that the fixation-related potentials may be important for the interpretation of results, but it remained unclear what exact analysis the reviewer had in mind. Therefore, we added a supplementary figure that shows the lambda waves, recorded from electrode Oz, descriptively in all experimental conditions. By binning the data by each trial's secondary saccade latency, the plot also shows how ERPs are influenced by secondary saccades, that is, the lambda response following the corrective eye movement. We reference this new figure in the main text where necessary, for instance when discussing the onsets of above-chance classification:

> Classification accuracy increased sharply around 50 ms after saccade offset, reaching a first peak at around 100 ms – the typical latency of the occipital λ response occurring after real and simulated eye movements (Riemslag et al., 1987; Ebersole and Galambos, 1973; Scott and Bickford, 1967; Scott et al., 1981; Dimigen et al., 2009), which was in the same way present in our data after both primary and secondary saccades (Figure A4).

R2.P4 2. Page 2, line 81: missing "of the trials" or "of the cases."

Response This omission has been corrected thanks to the reviewer's comment.

R2.P5 3. Page 2, line 92: saccade latencies — are they calculated relative to the first saccade offset or to stimulus onset?

Response We thank the reviewer for pointing out this inaccuracy. Throughout the manuscript, secondary saccade latency refers to the interval between primary saccade offset and secondary saccade onset. We added this detail as follows:

> Secondary saccade latency, here defined as the time elapsed between primary saccade offset and secondary saccade onset, varied considerably across target movement directions ...

References

Burr, D., Morrone, M., and Ross, J. (1994). Selective suppression of the magnocellular visual pathway during saccadic eye movements. *Nature*, 371(6497):511–513.

Dimigen, O., Valsecchi, M., Sommer, W., and Kliegl, R. (2009). Human microsaccade-related visual brain responses. *Journal of Neuroscience*, 29(39):12321–12331.

Duhamel, J.-R., Colby, C. L., and Goldberg, M. E. (1992). The updating of the representation of visual space in parietal cortex by intended eye movements. *Science*, 255(5040):90–92.

Ebersole, J. S. and Galambos, R. (1973). Lambda waves evoked by retinal stimulation in the absence of eye movements. *Electroencephalography and Clinical Neurophysiology*, 35(1):39–47.

Edwards, G., VanRullen, R., and Cavanagh, P. (2018). Decoding trans-saccadic memory. *Journal of Neuroscience*, 38(5):1114–1123.

Fabius, J. H., Fracasso, A., Acunzo, D. J., Van der Stigchel, S., and Melcher, D. (2020). Low-level visual information is maintained across saccades, allowing for a postsaccadic handoff between visual areas. *Journal of Neuroscience*, 40(49):9476–9486.

Moran, C., Johnson, P. A., Landau, A. N., and Hogendoorn, H. (2024). Decoding remapped spatial information in the peri-saccadic period. *Journal of Neuroscience*, 44(30).

Riemslag, F., Van Der Heijde, G., and Van Dongen, M. (1987). Are eye movement evoked potentials different from pattern reversal evoked potentials? *Documenta ophthalmologica*, 66:279–289.

Schweitzer, R. and Rolfs, M. (2020). Intra-saccadic motion streaks as cues to linking object locations across saccades. *Journal of Vision*, 20(4):17.

Scott, D. F. and Bickford, R. G. (1967). Electrophysiologic studies during scanning and passive eye movements in humans. *Science*, 155(3758):101–102.

Scott, D. F., Moffett, A., and Bickford, R. (1981). Comparison of two types of visual evoked potentials: pattern reversal and eye movement (lambda). *Electroencephalography and clinical neurophysiology*, 52(1):102–104.
